# Metabolite Profiling and Transcriptome Analyses Provide Insight Into Phenolic and Flavonoid Biosynthesis in the Nutshell of Macadamia Ternifolia

**DOI:** 10.3389/fgene.2021.809986

**Published:** 2022-02-21

**Authors:** Rui Shi, Liang Tao, Xinghao Tu, Chunsheng Zhang, Zhi Xiong, Abraham Rami Horowitz, Jiftah Ben Asher, Jun He, Faguang Hu

**Affiliations:** ^1^ Key Laboratory for Forest Resources Conservation and Utilization in the Southwest Mountains of China, Ministry of Education, International Ecological Foresty Research Center of Kunming, Horticulture and Landscape Architecture, Southwest Forestry University, Kunming, Yunnan, China; ^2^ Yunnan Institute of Tropical Crops, Xishuangbanna, Yunnan, China; ^3^ Key Laboratory of Hainan Province for Postharvest Physiology and Technology of Tropical Horticultural Products, South Subtropical Crops Research Institute, Chinese Academy of Tropical Agricultural Sciences, Zhanjiang, China; ^4^ Office of Academic Affairs, Yunnan University of Finance and Economics, Kunming, China; ^5^ French Associates Institute for Agriculture and Biotechnology of Dryland, The Jacob Blaustein Institutes for Desert Research, Ben-Gurion University of the Negev, Be’er Sheva, Israel; ^6^ State Key Laboratory of Component-based Chinese Medicine, Tianjin University of Traditional Chinese Medicine, Tianjin, China; ^7^ Institute of Tropical and Subtropical Cash Crops, Yunnan Academy of Agricultural Sciences, Baoshan, China

**Keywords:** macadamia, nutshell, metabolic, transcript, phenolic, flavonoid

## Abstract

Macadamia ternifolia is a dynamic oil-producing nut crop in the world. However, the nutshell is frequently considered as a low-quality material. Further, its metabolic profile is still uncharacterized. In order to explore the industrial significance of the nutshell, this study performed metabolic and transcriptomic analyses at various developmental stages of the nutshell. The qualitative and quantitative metabolic data analysis identified 596 metabolic substances including several species of phenolic acids, flavonoids, lipids, organic acids, amino acids and derivatives, nucleotides and derivatives, alkaloids, lignans, coumarins, terpenoids, tannins, and others. However, phenolic acids and flavonoids were predominant, and their abundance levels were significantly altered across various developmental stages of the nutshell. Comparative transcriptome analysis revealed that the expression patterns of phenolic acid and flavonoid pathway related genes were significantly changed during the nutshell growth. In particular, the expression of phenylalanine ammonia-lyase, C4H, 4CL, CHS, CHI, F3H, and FLS had dynamic differences at the various developmental stages of the nutshell. Our integrative metabolomic and transcriptomic analyses identified the key metabolic substances and their abundance levels. We further discussed the regulatory mechanism of phenolic and flavonoid biosynthesis in the nutshell of M. ternifolia. Our results provide new insights into the biological profiles of the nutshell of M. ternifolia and help to elucidate the molecular mechanisms of phenolic and flavonoid biosynthesis in the nutshell of M. ternifolia*.*

## Introduction

Macadamia nuts belong to the Proteaceae family, native to Australia, and are considered one of the highest oil quality nuts in the world (Mast et al., 2008; Hu et al., 2019). It is commercially cultivated in China, Hawaii, South Africa, New Zealand, Kenya, and several South American countries (Rengel et al., 2015; Navarro and Rodrigues, 2016). There are nine species in the Macadamia genus but only four of them have a wider public acceptance. In particular, Macadamia *integrifolia, and M. tetraphylla* had a large scale commercial production than *M. jansenii* and *M. ternifolia* (*Howlett et al., 2015*). The fruit of Macadamia contains a delicate and delicious cream to white colored kernel which covers in overall a thick dark green colored nutshell (Mast et al., 2008). The large part of the fruit is nutshell that is commonly wasted, and sometime used as low-quality raw material (O’Hare et al., 2019). The edible Macadamia nut contains essential nutrients, vitamins, minerals, and monounsaturated fatty acids (Malvestiti et al., 2017). Oleic and palmitoleic acids are predominately present in higher amounts in Macadamia nuts oil compared to other nut crops (Kaijser et al., 2000; Hu et al., 2019). Because of the high nutritive characteristics, Macadamia products are thought to be effective in medical field against cardiovascular diseases like myocardial infarction, cancer, arteriosclerosis, and dyslipidemia (Garg et al., 2003; Wang and Hu, 2017). In addition, Macadamia is also used in cosmetic industry to produce various skincare and anti-aging creams (Rafia, 2013). Macadamia *is largely cultivated in Australia* and approximately 35,000 tons of the shell is produced alone in Australia (Fan et al., 2018). For the environmental protection, there is a need to change the high amount of macadamia nutshell into useful by products to mitigate industrial based waste management problems.

The nutshell is very important in dry fruits. It synthesizes and transports essential nutrients to the kernel during stressful growth periods. Moreover, it not only regulate the fruit shape and size but also prevents mechanical and chemical injuries (Francoz et al., 2018). The Macadamia nutshell has an exceptional cell wall thickness which is considered as an isotropic wood by some researchers (Wang and Mai, 1994). Some researches described that two or three different layers with lignified cells are present in the nutshell which increases the hardness of the wall (Hartung and Storey, 1939). Macadamia nutshell is widely used as a direct burning material. It is utilized as a compost to prevent weed growth and moisture loss (Conesa et al., 2000; Girdis et al., 2017). In addition, Macadamia nutshell is used to produce biomass pyrolysis through controlled high-temperature serial reactions (Poinern et al., 2011). Several pyrolysis-based biochar applications include steel production (Kumar et al., 2017), soil amendments (Wrobel-Tobiszewska et al., 2018), and de-nitrification mechanism (Bakly et al., 2019). Similarly, the nutshells of almond, hazelnut, walnut, and apricot are a cost-effective source of activated carbons for treatment of industrial effluents (Aygün et al., 2003). Interestingly, extracts from Macadamia nutshell have been widely utilized to develop fragrance, hair, and skincare products in recent years (Syed and Walele, 2006). However, research in Macadamia has been largely focused on the physical, biological, and health properties of the kernel oil (Akhtar et al., 2006; Garg et al., 2007). Limited information is available about the chemical composition of the active metabolic compounds of the nutshell. There is a need of increased knowledge to synthesize high-quality by-products from Macadamia nutshell.

In recent years, multi-omic analyses such as the integration of transcriptomic and metabolomic data have gained much importance to investigate the regulatory mechanism of metabolite substances in plants (Chen et al., 2014; Shen et al., 2021). In this regard, mass spectrometry has been broadly applied to analyze the composition and concentration of metabolic substances (Matsuda et al., 2009; Fang et al., 2019). Whereas, transcriptome sequencing provides effective means to unveil gene to metabolite networks in oilseed crops (Zhao et al., 2018; Wu et al., 2020). Previous high-throughput research already characterized the biological mechanism of different metabolites such as fatty acids, sugars, amino acids, phenolic acids, and flavonoids biosynthesis in various oilseed crops (Bourgis et al., 2011; Alagna et al., 2012; Zheng et al., 2017; Rehman et al., 2018; Zhang et al., 2019). The key genes that regulate the biosynthesis and degradation of key metabolites have been uncharacterized in the nutshell of *M. ternifolia*. To accomplish this research gap, our research group performed a comprehensive metabolome and transcriptome analyses at various developmental stages of the nutshell. Our study identified the metabolic substances, characterized their abundance, and established biological profiles of key metabolites in *M. ternifolia* nutshell. These results will be important to improve the existing knowledge allied with qualitative and quantitative metabolic substances of the nutshell. Furthermore, it provides an important theoretical reference for future research which aims to investigate the molecular mechanism of polyphenol biosynthesis and a better valorization of the nutshell of *M. ternifolia*.

## Results

### Metabolite Composition and Concentration in *M. Ternifolia* Nutshell

**FIGURE 1:**
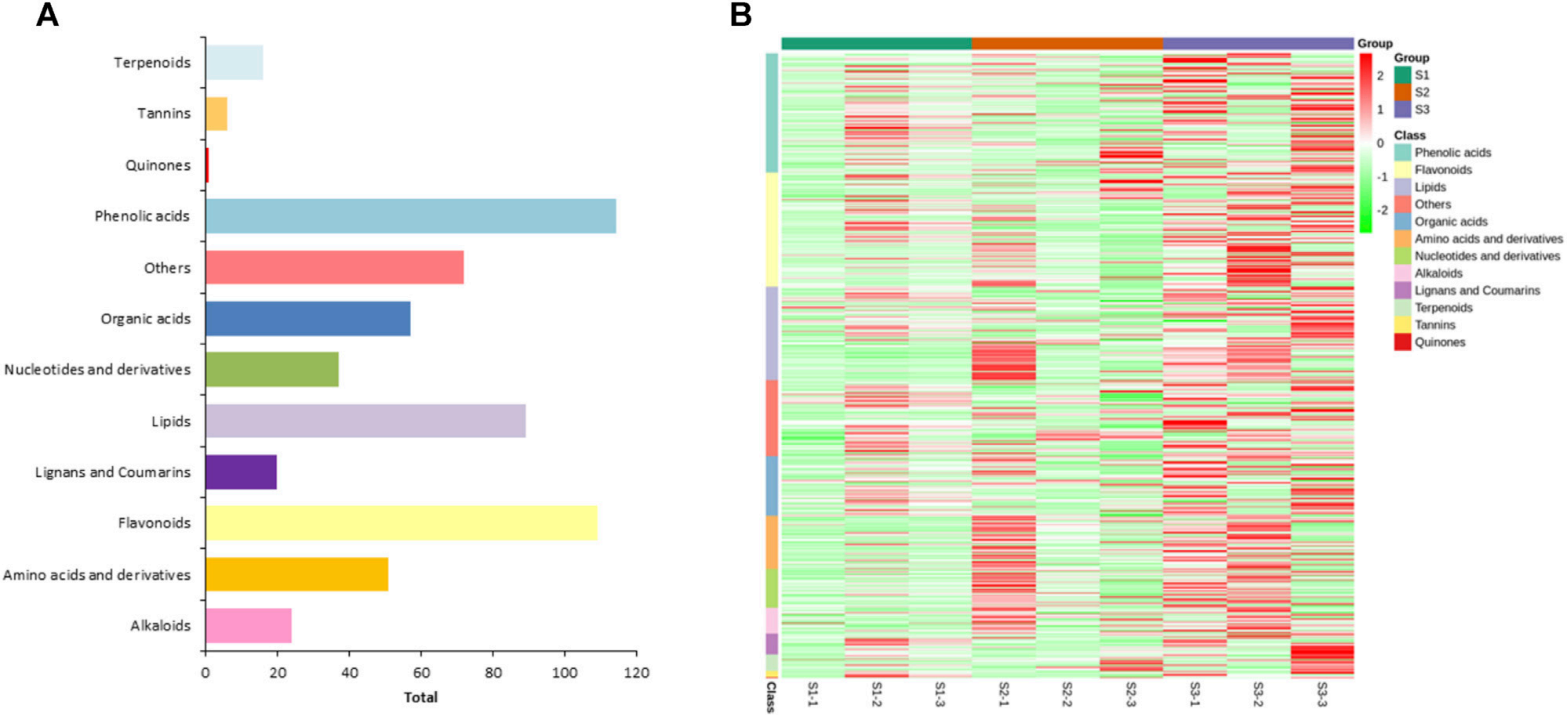
Classification and abundance of the metabolites at three developmental stages of M. ternifolia nutshell. **(A)** The classification results of different metabolites **(B)** The abundance level of different metabolites.

**FIGURE 2:**
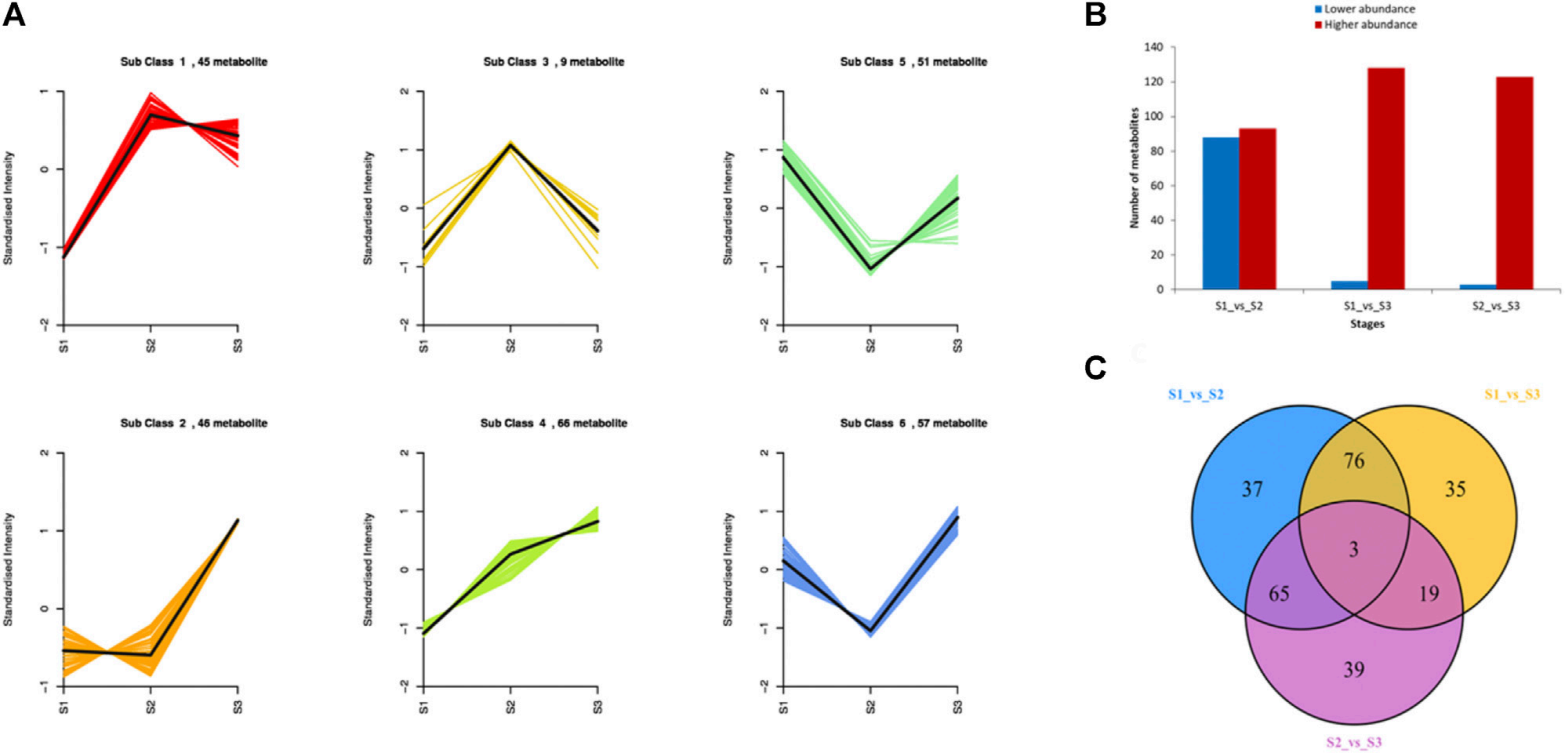
The K-means clustering and statistics of differential metabolites at three developmental stages of *M. ternifolia* nutshell **(A)** The clustering results of metabolites **(B)** Total number of up regulated and down regulated metabolites **(C)** The distribution of differential metabolites at various development stages.

To explore the metabolic profiles of *M. ternifolia* nutshell, the nutshell samples were selected at the stages of young fruit (S1), medium fruit (S2), and mature fruit (S3). A total of 596 metabolites were identified which were categorized into 12 different classes. The major metabolites were phenolic acids, flavonoids, and lipids followed by other classes. For example, there were 114 phenolic acids, 109 flavonoids, 89 lipids, 72 others, 57 organic acids, 51 amino acids and derivatives, 37 nucleotides and derivatives, 24 alkaloids, 20 lignans, and coumarins, 16 terpenoids, 6 tannins, and 1 quinone (Figure 1A). All metabolites had distinct abundance at various development stages of a nutshell (Figure 1B). Specifically, many metabolites exhibited a higher abundance at the mature fruit stage as compared to early stages. The metabolites associated with different types of benzoic acid, coumaroylquinic acid, and chlorogenic acid were key phenolic acids (Supplementary Table S1). Among flavonoids, the different forms of methoxyflavone, glucuronideflavone, glucosideflavone, and rutinosideflavone had most abundance. The metabolites in the lipid class were mainly decenoic acid, palmitic acid, linolenic acid, and linolenoyl glycerol. Cluster analysis arranged the 275 metabolites into six major classes. Each class was based on the metabolic abundance during the different stages of nutshell development (Figure 2A). Classes 1 and 3 contained 45 and 9 metabolites, respectively, with a higher abundance at the S2 stage of the nutshell. Class 5 had 51 metabolites that showed the highest abundance at the S1 stage, whereas classes 2, 4, and 6 contained 45, 56, and 57 metabolites, respectively. All metabolites of these classes exhibited the highest abundance during the S3 stage. These results indicated that phenolic acids and flavonoids are predominant metabolites in the nutshell. In addition, metabolite composition and concentration changed during the developmental stages of nutshell in *M. ternifolia*.

### Differential Metabolites Analysis in *M. Ternifolia* Nutshell

The abundance of each metabolite was compared among growth stages to identify differential metabolites. There were 181 differentially accumulated metabolites in the comparison of S1 and S2, 88 of which showed a lower abundance and 93 showed a higher abundance at S1 than S2 (Figure 2B). In the comparison of S1 and S3, 133 metabolites showed a significantly different accumulation. Of which, only 5 metabolites showed a decreased abundance and 128 metabolites showed a higher abundance at S1 as compared to S3. A total of 126 metabolites had a significant difference between the comparison S2 and S3. Relative to S3, only 3 metabolites showed lower abundance and 123 metabolites showed a higher abundance at the S2 stage. Venn diagram revealed that a major portion of the metabolites was overlapped among the different stages, whereas few metabolites were stage-specific (Figure 2C). For example, only 37, 35, and 39 metabolites were specific to S1vsS2, S1vsS3, and S2vsS3, respectively. As expected, metabolites related to phenolic acids, flavonoids, lipids, and amino acid derivatives had dynamic variations among the different stages. The metabolites belonging to these classes exhibited significantly higher abundance at the late growth stage than early growth stages (Supplementary Figure S1). Overall, *M. ternifolia* displays significant metabolic differences at the different stages of nutshell growth. In particular, different species of phenolic acids and flavonoids were found with dynamic changes.

### Identification of Key Metabolites in *M. Ternifolia* Nutshell

**FIGURE 3:**
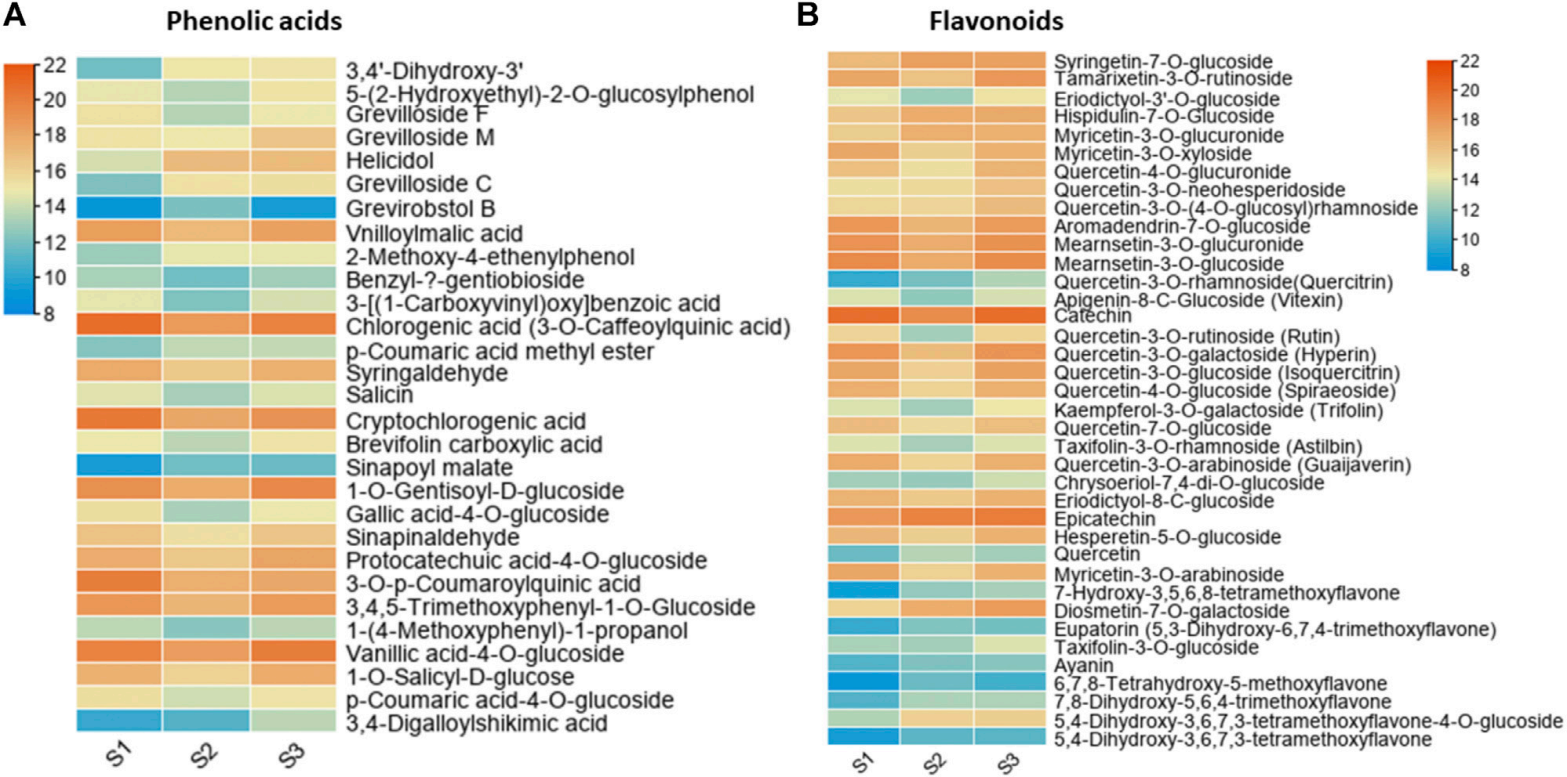
Heatmaps of key metabolite substances at three developmental stages of *M. ternifolia* nutshell **(A)** Phenolic acids **(B)** Flavonoids.

The results of metabolic composition and profiling revealed many metabolites with differential abundance in nutshell. To identify putative key metabolites which are critical components of the nutshell, we further selected those metabolites which showed significant abundance throughout various development stages of the nutshell. In this way, a total of 67 highly differential abundant metabolites were identified (Figure 3). A total of 29 metabolites belong to phenolic acids and 38 to flavonoids. It was observed that vanillic acid 4-O-glucoside, 3-O-*p*-coumaroylquinic acid, syringaldehyde 1-O-gentisoyl-D-glucoside, 3,4,5 trimethoxyphenyl-1-O-glucoside, chlorogenic acid (3-O-caffeoylquinic acid), cryptochlorogenic acid, vnilloylmalic acid, and protocatechuic acid-4-O-glucoside were major the phenolic components of the nutshell (Figure 3A). However, vanillic acid 4-O-glucoside, chlorogenic acid (3-O-caffeoylquinic acid), cryptochlorogenic acid, 3-O-*p*-coumaroylquinic acid, and syringaldehyde were detected in a very high abundance. While benzyl-β-gentiobioside, sinapoyl malate, and grevirobstol-B had the lowest abundance among all identified phenolic acids. Quercetin-linked glucoside related flavonoids were the major components of the nutshell, which exhibited dynamic differences among different developmental stages (Figure 3B). However, catechin, epicatechin, mearnsetin-3-O-glucoside, mearnsetin-3-O-glucuronide, and quercetin-3-O-galactoside showed the highest abundance among all identified flavonoids. In contrast, ayanin, eupatorin (5,3-dihydroxy-6,7,4-trimethoxyflavone), 5,4 dihydroxy-3,6,7,3-tetramethoxyflavone, and 6,7,8-tetrahydroxy-5-methoxyflavone had lower abundances at all stages of nutshell growth in this study. These results suggest that different classes of phenolic acids and flavonoids linked with glucosides are the primary components of nutshell in *M. ternifolia*.

### Ribonucleic Acid Sequencing and Unigenes Functional Annotations

The nutshell samples including S1, S2, and S3 were RNA-sequenced to explore the gene expression changes associated with regulation of different metabolites in *M. ternifolia* nutshell. According to the results of the RNA-sequencing, on average 45,282,444, 53,082,622, and 50,630,658 raw reads were acquired for S1, S2, and S3, respectively (Table 1). More than 7 GB of clean data were generated after removing low-quality reads for each sample. The Q20 score was greater than 98% and Q30 was 94% in our study, whereas the guanine-cytosine (GC) content was almost 45% for all samples. A total of 20,8119 unigenes were assembled with Trinity software (Trinity release v2.4.0, United States) from clean reads with a mean length of 1,256, N50 length of 1,883 bp, and N90 length of 569 bp (Supplementary Table S2). These unigenes had a length distribution range from 200 to >2,000 bp. A total of 16,565 unigenes had a length range from 200 to 300 bp, 21,579 unigenes had a length range from 300 to 400 bp. However, most unigenes had a length range >2,000 bp (Supplementary Figure S2A). The blast results revealed that the majority of unigenes had functional annotation within NR and Trembl databases followed by GO, Pfam, KEGG, SwissProt, and KOG databases (Supplementary Figure S2B).

**TABLE 1:** Summary of transcriptome analysis for three developmental stages of *M. ternifolia* nutshell.

Sample	Raw reads	Clean reads	Clean base (G)	Mapped reads (%)	Q20 (%)	Q30 (%)	GC content (%)
S1-1	44,743,254	42,558,824	6.4	88.2	98.5	95.4	45.5
S1-2	44,850,052	43,127,864	6.5	86.8	98.5	95.1	45.1
S1-3	46,254,028	44,371,420	6.7	86.8	97.4	92.8	45.2
S2-1	47,542,148	45,114,368	6.8	87.4	98.5	95.3	45.4
S2-2	46,886,012	45,060,870	6.8	86.5	98.5	95.3	45.3
S2-3	64,819,706	61,361,656	9.2	87.3	98.6	95.4	45.3
S3-1	48,965,352	47,067,132	7.1	86.3	97.5	93.1	45.2
S3-2	46,881,180	44,977,126	6.8	87.1	98.6	95.5	45.3
S3-3	56,045,442	53,501,474	8.0	86.9	98.6	95.4	45.3

### Differential Gene Expression and Functional Enrichment Analysis

**FIGURE 4:**
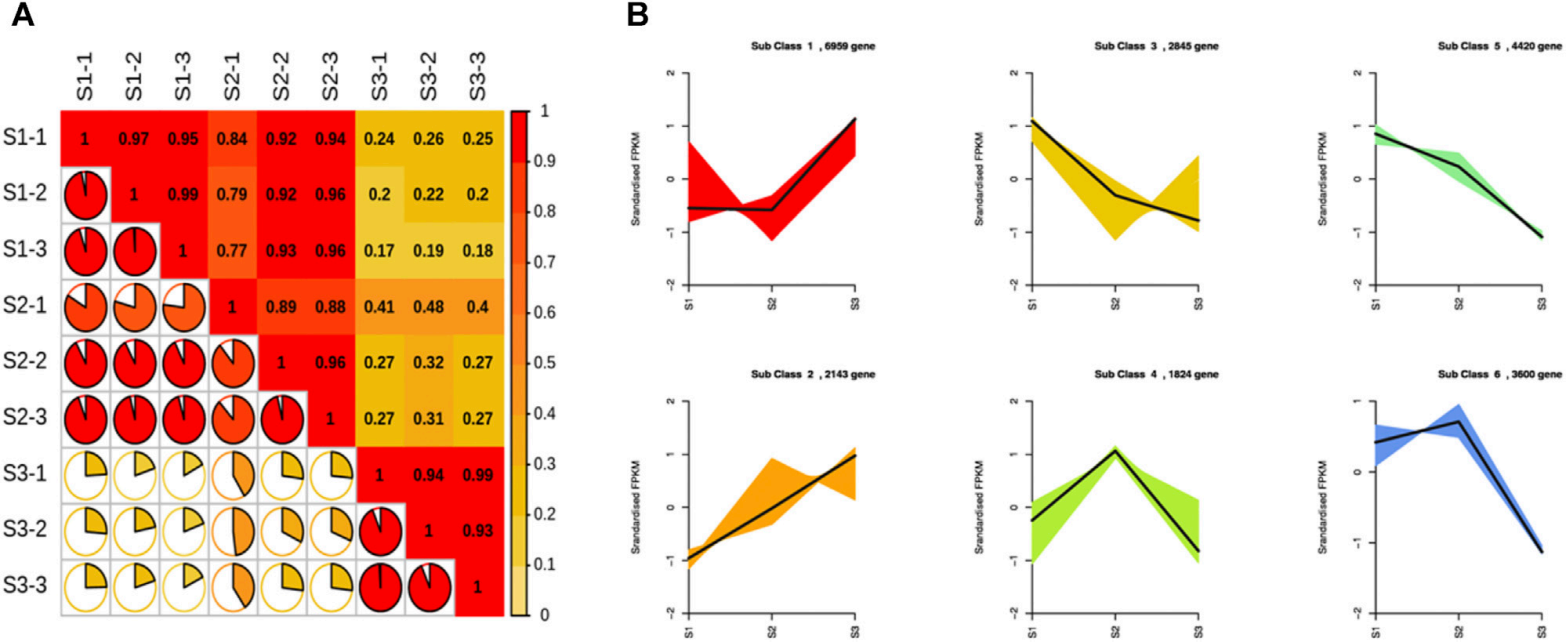
Correlation analysis and K-means clustering of unigenes at three developmental stages of *M. ternifolia* nutshell **(A)** Correlation analysis **(B)** K-means clustering of unigene.

The expression level of each unigene was measured in the form of FPKM. There was a strong correlation among the biological replicates. In contrast, S3 samples had a weak correlation with different repeats of S1 and S2 stages (Figure 4A). The correlation coefficient among different repeats was almost greater than 0.95 for each stage, while S1 and S2 repeats had a correlation coefficient of 0.25 with the S3 stage. The gene expression-based clustering analysis showed distinct gene expression profiles across S1, S2, and S3. Cluster analysis divided the expression profiles of unigenes into six major classes (Figure 4B). Classes 1 and 2 contained 6,959 and 2,143 unigenes, respectively, characterized by a high expression at the S3 stage. Classes 3, 4, 5, and 6 contained 2,845, 1,824, 4,420, and 3,600 unigenes, respectively. All unigenes of these classes exhibited a lower expression during the S3 stage than the other two stages of nutshell growth. In addition, unigenes belonging to classes 3 and 5 exhibited a higher expression at the S1 stage compared to others, whereas classes 4 and 6 contained unigenes with a higher expression at the S2 stage.

**FIGURE 5:**
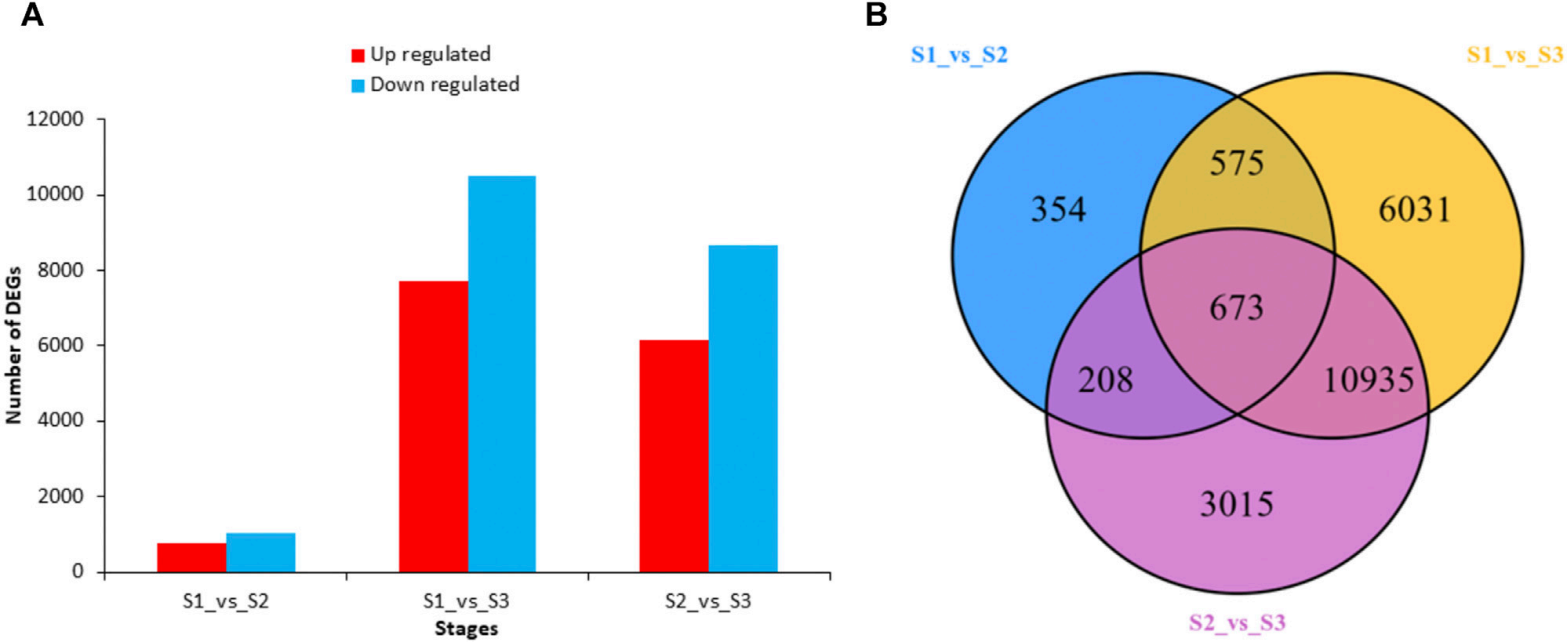
The total number of up-regulated and down-regulated DEGs and their distribution at three developmental stages of *M. ternifolia* nutshell **(A)** Total number of up-regulated and down regulated DEGs **(B)** The distribution of DEGs among the developmental stages.

**FIGURE 6:**
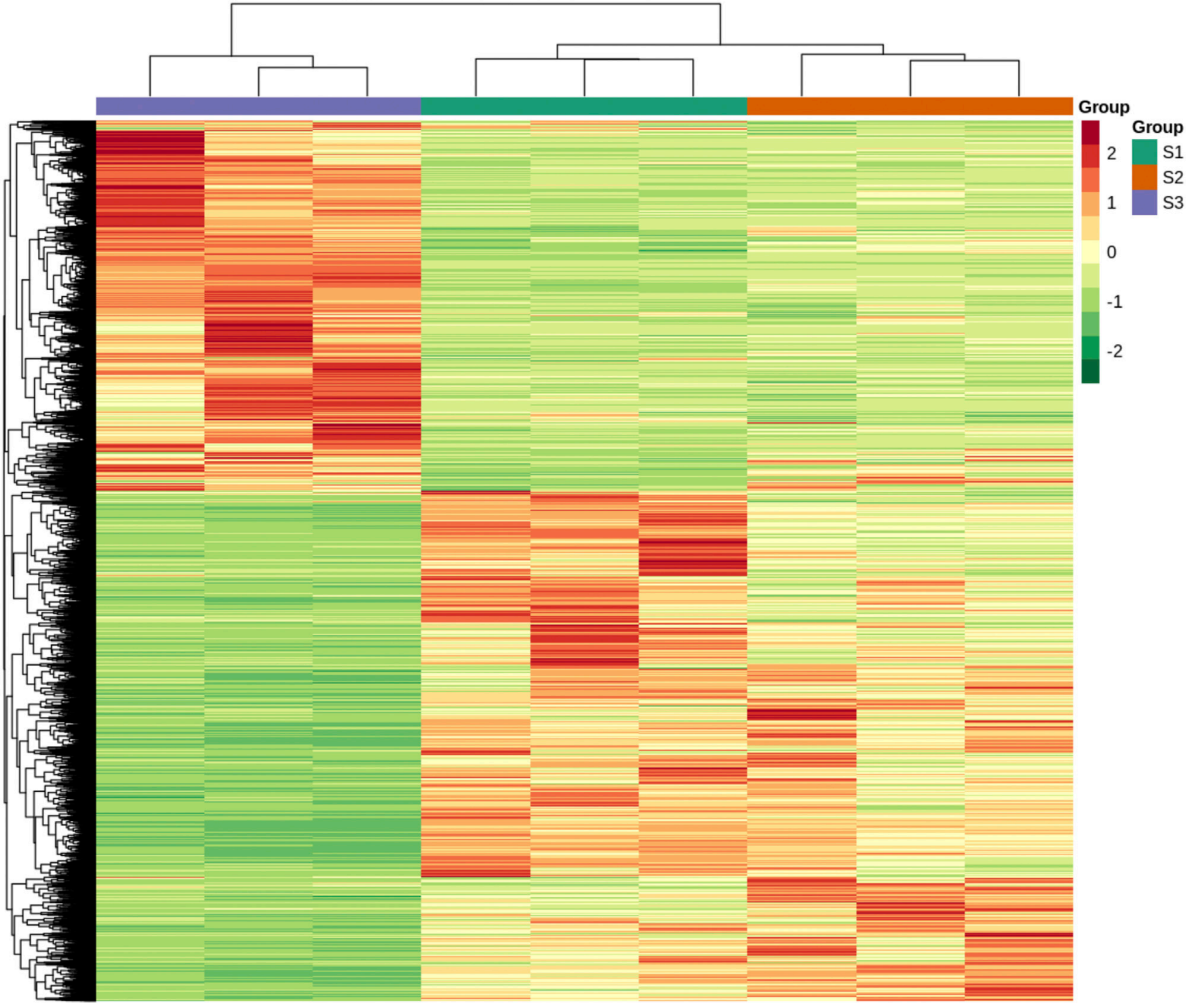
All DEGs expression heatmap at three developmental stages of *M. ternifolia* nutshell.

To investigate the gene expression differences between the three developmental stages, we made pairwise comparison of the transcript profiles in S1, S2, and S3. The DEGs were considered significant if they had log2 Fold Change ≥1 or ≤ -1 and the false discovery rate <0.05. Comparative analysis showed a total number of 1,810 differentially expressed genes (DEGs) in S1vsS2 (Figure 5A). In S1vsS3, there were 18,214 DEGs in total, which stated much higher expression differences among these stages. Comparative analysis in S2vsS3 revealed a total of 14,831 DEGs. The Venn diagram distribution revealed that a total of 21,791 DEGs existed across the three development stages (Figure 5B). There were only 673 overlapped DEGs between the three stages. However, the comparison of S1vsS3 and S2vsS3 had 10,935 overlapped DEGs. All DEGs expression heatmap stated an undulating expression trend at different developmental stages. But, most DEGs were found with higher expressions level at S1 and S3 (Figure 6). Functional characterization showed that metabolic and biosynthesis of secondary metabolites-related pathways had more significance in comparisons of S1vsS2, S1vsS3, and S2vsS3 (Supplementary Figure S3). However, S1vsS3 and S2vsS3 had almost more than 2,000 metabolic DEGs, whereas the metabolic DEGs between S1 and S2 were only 300. Theoretically, we deduce that a large number of metabolic DEGs with undulating expression profiles are associated with the differences in metabolite abundances/composition observed at the three developmental stages of nutshell in *M. ternifolia*.

### Identification of Key Genes Involved in Phenolic Acids and Flavonoids Related Metabolism

**FIGURE 7:**
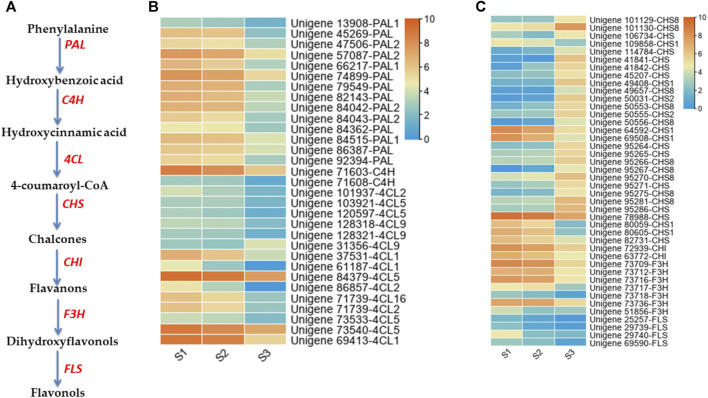
Expression profiles of phenolic acid and flavonoid pathway genes distribution at three developmental stages of *M. ternifolia* nutshell **(A)** Simplest diagram of phenolic acid and flavonoid biosynthesis pathway **(B)** Phenolic acid metabolism related DEGs **(C)** Flavonoids pathway related DEGs.

Our metabolomics results showed that phenolic acid is the major component of nutshell in *M. ternifolia.* Therefore, genes with significant expression differences across stages and involved in the regulation of phenolic acids metabolism were excavated. The phenylalanine is essential to produce phenolics and flavonoids metabolites via different enzymes including phenylalanine ammonia-lyase (PAL), cinnamate-4-hydroxylase (C4H), 4-coumaroyl coenzyme A ligase (4CL), chalcone synthase (CHS), chalcone isomerase (CHI), flavanone -3-hydroxylase (F3H), and flavonol synthase (FLS) (Figure 7A). In our study, 73 genes annotated with phenolic and flavonoids metabolites exhibited significant expression differences at different developmental stages of the nutshell (Figures 7B,C). Specifically, the expression of 14 *PAL uni*genes (*unigene 13,908-PAL1, unigene 45269-PAL, unigene 47,506-PAL2, unigene 57,087-PAL2, unigene 66,217-PAL1, unigene 74899-PAL, unigene 79549-PAL, unigene 82143-PAL, unigene 84,042-PAL2, unigene 84,043-PAL2, unigene 84362-PAL, unigene 84,515-PAL1, unigene 86387-PAL,* and *unigene 92394-PAL*) had a significant lower expression at S2 and S3 (Figure 7B). Similarly, *unigene 71,603-C4H* and *unigene 71,608-C4H* showed a lower expression at late stages of nutshell growth. A total of 15 unigenes were identified to encode *4CL*, including three for *4CL1*, three for *4CL2*, five for *4CL5*, three for *4CL9*, and one for *4CL16*. The expression profiles of all these 4CL genes were peaked during the early stages (Figure 7B).

Interestingly, there were 11 *CHS* unigenes with significant differential expression patterns across the three stages of nutshell growth. In particular, *unigene 95286-CHS* had a strong expression at the late stage (Figure 7C). In contrast, *unigene 78988-CHS* showed much high expression during early stages. Among other identified *CHS* encoding unigenes, there were 6 for CHS1, two for *CHS2,* and ten for *CHS8.* The expression trends of two genes denoted as *unigene 72939-CHI and unigene 63772-CHI* exhibited up regulated expression at the early stage compared to the late stage. The *F3H* and *FLS* enzymes encoding genes are vital to synthesize different kinds of flavonoids. The expression of seven *F3H* genes including *unigene 73,709-F3H, unigene 73,712-F3H, unigene 73,716-F3H, unigene 73,717-F3H, unigene 73,718-F3H, unigene 73,736-F3H,* and *unigene 51,856-F3H* showed peak expression at S1. Similarly, four *FLS* enzymes encoding genes such as *unigene 25257-FLS*, *unigene 29739-FLS*, *unigene 29740-FLS*, and *unigene 69590-FLS* had remarkably low expression levels at late stages of nutshell growth (Figure 7C). The dynamic expression changes in phenolic acid and flavonoid pathway genes indicate their critical role in the regulation of metabolites in the nutshell of *M. ternifolia*.

### qRT-Polymerase Chain Reaction Validation of Key Genes Related to Polyphenol Compounds

**FIGURE 8:**
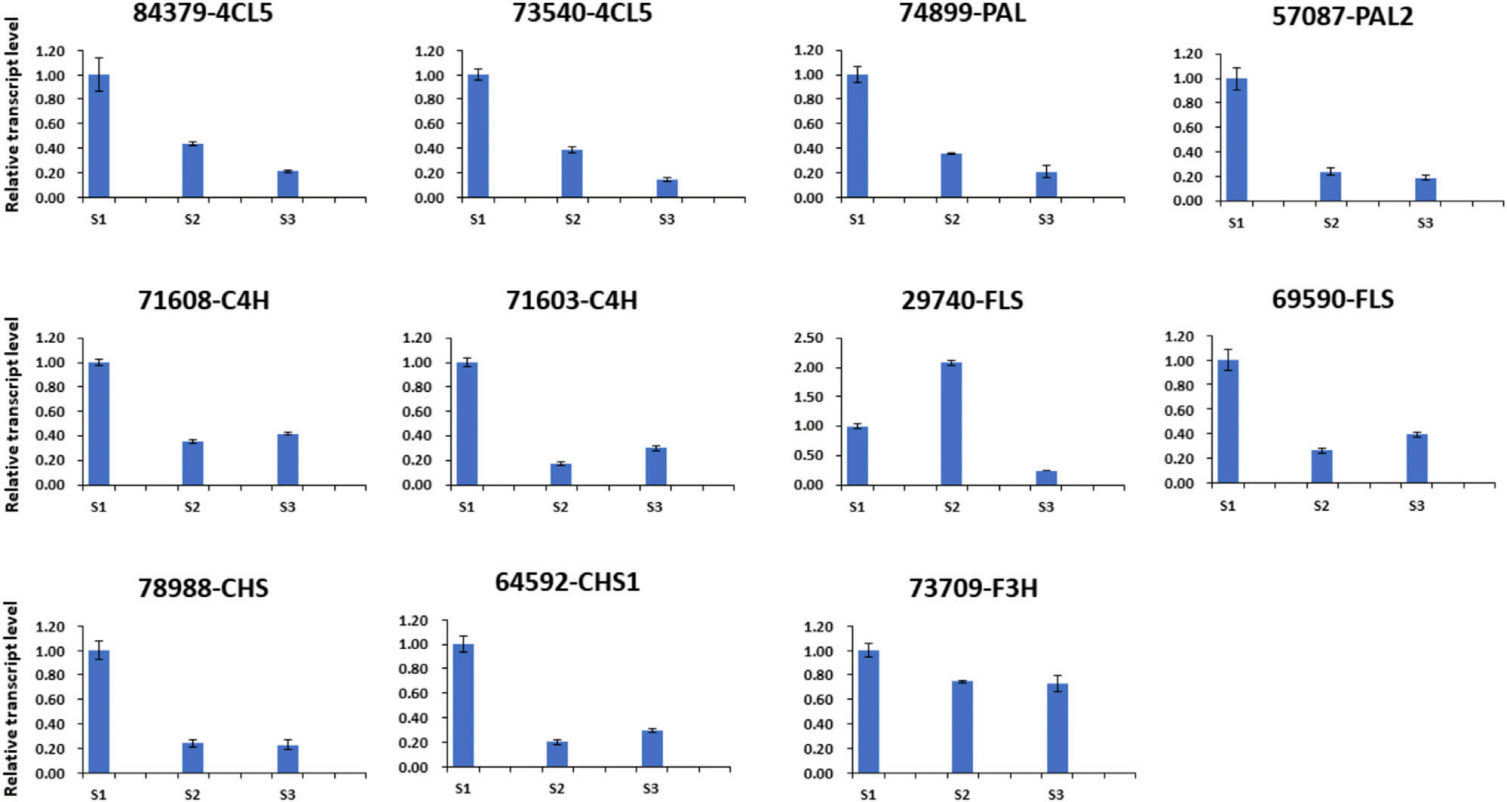
The qRT-PCR of polyphenol compounds related genes at three developmental stages of nutshell.

Based on the metabolic and transcriptome analysis, 11 genes (*unigene 57,087-PAL2, unigene 74899-PAL, unigene 71,603-C4H, unigene 71,608-C4H, unigene 84,379-4CL5, unigene 73,540-4CL5, unigene 64,592-CHS1, unigene 78988-CHS, unigene 73,709-F3H, unigene 29740-FLS*, and *unigene 69590-FLS*) involved in polyphenol compounds metabolism were selected to perform a qRT-PCR analysis. All genes showed a dynamic expression difference at the three developmental stages of the nutshell (Figure 8). These results speculated that these genes most likely play important role in metabolites accumulation in the nutshell of *M. ternifolia*. Furthermore, these findings validate our transcriptome data analysis.

## Discussion

### Polyphenol Compounds Profiles in the Nutshell of *M. Ternifolia*

In *Macadamia* nuts, the kernel is covered in a hard nutshell and considered as a premium product. It has been utilized to produce healthy diet for humans. However, the nutshell is still not well valorized in *Macadamia* nuts. Our study showed that *M. ternifolia* had a diversity of metabolic compounds in the nutshell with polyphenols especially phenolic acids and flavonoids being the major metabolites present in the nutshell. In addition, both the composition and concentration of these metabolites significantly changed at the three developmental stages of the nutshell. These functional metabolites may have useful applications in medical and ornamental industries. In plants, phenolic acids and flavonoids are low-molecular-weight pervasive groups (Ghasemzadeh and Ghasemzadeh, 2011; Rehman et al., 2018). Phenolic compounds are carbon-based secondary metabolites, which act as powerful antioxidants, antiviral, and anti-allergic agents (Yagi et al., 2013; Rouphael et al., 2017). They can absorb free radical oxygen, which subsequently reduced aging, heart attack, and cancer diseases (Hollman, 2001). Additionally, environmental factors such as light, temperature, nutrient availability, and salinity affected the abundance of phenolic compounds in plants (Marin et al., 2015; Sgherri et al., 2017; Henry-Kirk et al., 2018). It has been stated that the biosynthesis of different types of phenolic compounds play a vital role against pathogen and insect defense (Beckman, 2000). In this regard, increased information about phenolic profile can be important to mitigate anthracnose husk rot disease, which is a major threat to *Macadamia* nut production worldwide (Campbell, 2015). In *M. ternifolia*, glucosides and chlorogenic acid derived phenolic acids had a dynamic abundance in nutshell. A previous research has clarified that chlorogenic acid is characterized by antioxidant and anti-inflammatory properties (Sasaki et al., 2010). Therefore, it serves as a precursor in therapeutics, cosmetics, and food industries. In addition, phenolic acids are useful to enhance the organoleptic characters of food items, reduce skin damage, and avoid itching (Croft, 1998; Tan, 2000). Flavonoids in plants belong to polyphenols, synthesized by aromatic rings, and classified into different kinds of flavones, flavanones, chalcones, isoflavones, flavonols, deoxy-flavonoids, and anthocyanins (Panche et al., 2016). They have been widely applied in food, pharmaceutical, medicinal, and cosmetic industries. Almost every group of flavonoids has potential health benefits through antioxidant defense mechanisms (Nijveldt et al., 2001). Interestingly, flavonoids were major component in the nutshell of *M. ternifolia*. In particular, quercetin linked glucoside, catechin, epicatechin, mearnsetin-3-O-glucoside, mearnsetin-3-O-glucuronide, and quercetin-3-O-galactoside had the highest abundance among all the identified flavonoids. The stone fruits such as peach, sweet cherry, apricot, plums, and almond skin have a diversity of polyphenols. However, catechin, epicatechin, chlorogenic acid derivatives are the most common among detected polyphenols in these fruits (Garrido et al., 2008; Campbell and Padilla-Zakour, 2013; Pinu, 2016; Koprivica et al., 2018). Previous research revealed several health-promoting benefits of polyphenols obtained from plants (Lara et al., 2020). It is noteworthy that polyphenols by-products obtained from almond skin is used to reduce viral, inflammatory, allergic, mutagenic, and carcinogenic disorders (Siriwardhana and Shahidi, 2002). Improved knowledge about bioactive compounds present in the nutshell of *M. ternifolia* could be useful to produce several kinds of antioxidant materials, which can be utilized in the food industry and could also be used as functional ingredients in pharmaceutical and cosmetic industries.

### Regulatory Mechanism of Polyphenols in the Nutshell of *M. Ternifolia*

The phenolic compounds are mainly synthesized from the shikimate/phenylpropanoid pathway in plants (Tzin and Galili, 2010; Vogt, 2010). In this pathway, phenylalanine act as a precursor of different kinds of phenolic acids and flavonoids (Weisshaar and Jenkins, 1998). Nearly, all genes involved in the phenylpropanoid pathway has been investigated in *Arabidopsis,* poplar, and rice (*Hamberger et al., 2007*)*.* In brief, phenylalanine in the first step generates derivatives of hydroxybenzoic acid by the action of the *PAL* gene family. Later, serial reactions regulated by *C4H* and *4CL* gene families synthesized various forms of gallic acids, cinnamic acids, caffeic acids, ferulic, acids, sinapic acids, and chlorogenic acids. In the nutshell of *M. ternifolia,* a dynamic expression of 14 *PAL*, 2 *C4H,* and 15 *4CL* genes may regulate the composition of different phenolic acids (Ragg et al., 1981; Chen et al., 2007; Chang et al., 2008). Additionally, the expression of *PAL, C4H,* and *4CL* genes is altered significantly in response to growth conditions, abiotic and biotic stresses (Lee et al., 1995; Bellés et al., 2008; Huang et al., 2010). Flavonoids are synthesized from branches of the phenylpropanoid pathway (Dixon and Steele, 1999). *CHS* genes catalyzed the first committed step to generate chalcone, which is a primary precursor of all flavonoids. In the next step, chalcones are isomerized into flavanones by the action of *CHI* genes. The gene family *F3H* generates dihydroflavanols from flavanones, which finally form flavonols by the action of FLS genes. Several genes allied with *CHS, CHI,* and *FLS* showed significantly altered expression in developing nutshell of *M. ternifolia.* These results predict that flavonoids pathway genes expression and the abundance of flavonoids compounds are most likely interlinked with each other. A recent study in *Brassica juncea* revealed a strong correlation between flavonoids pathway genes expression and the abundance of flavonoids compounds (Shen et al., 2021). Flavonoids pathway genes are well understood in *Arabidopsis (* *Saito et al., 2013* *), brassica (* *Liu et al., 2013* *),* and pear (Feng et al., 2010). Transgenic studies reported that the phenylpropanoid pathway is comprised of several complex chemical reactions. The increased or decreased expression of a specific gene may not lead to a clear phenotype, which is probably due to the synthesis of the certain compound via an alternative pathway (Sewalt et al., 1997). However, a strong correlation between metabolite and transcript profiling of specific compounds is critical to identify a single gene by following advance transgenic quantifications (Rohde et al., 2004). Because the genetic transformation protocol of *M. ternifolia* is not yet well established, it is not possible at this stage to study the functions of some candidate genes identified in this study. Nonetheless, our integrative metabolome and transcriptome analysis are effective to identify key potential genes responsible for polyphenols biosynthesis in the nutshell of *M. ternifolia.*

## Materials and Methods

### Plant Materials and Analysis of Metabolic Substances

Plant materials used in this study were taken from *M. ternifolia.* The nutshell samples were collected in three biological replicates at the young fruit (S1), medium fruit (S2), and mature fruit (S3) stages. The harvested samples were placed immediately in liquid nitrogen and stored in an ultra-low temperature refrigerator at −80°C for metabolomics and transcriptomics analysis. In order to explore the differences in metabolites in different samples, 1 g biological sample was ground into powder. Then 100 mg of powder was dissolved in 1.2 ml 70% aqueous methanol. Later on, samples were vortex for 30 min and saved into the refrigerator at 4°C overnight. The samples were centrifuged at 12000 rpm for 10 min. The upper clear liquid was collected in a sample bottle, filtered with a micro-porous membrane (0.22 um pore size), and stored for UPLC-MS/MS analysis. The ultra-performance liquid chromatography (UPLC) (Shimadzu Nexera X2) and tandem mass spectrometry (MS/MS) (Applied Biosystems 4500 QTRAP) were used in this study to perform metabolic analysis. The instrument running parameters and data acquisition methods were followed as previously described (Yin et al., 2019). Based on the local metabolic databases e.g., MzCloud, Massbank, Metlin, HMDB, and KEGG, qualitative and quantitative analysis of the metabolites for each sample was carried out. The identification of significantly differential metabolites was achieved with variable importance in projection (VIP) value combined with fold change (FC). The metabolites with FC ≤ 0.5 or ≥0.5 and VIP ≥1 were generally considered to be significant among the control group and the experimental group. The metabolites that differed more than 2 times or less than 0.5 were considered significant high or low differences between the control and experimental groups.

### Ribonucleic Acid Sequencing and Downstream Analysis

High quality total RNA were extracted with the CTAB method (Gambino et al., 2008) from samples of young (S1), medium (S2), and mature fruit (S3) nutshell of *M. ternifolia.* The Agarose gel electrophoresis was used to analyze the integrity of RNA and the presence of contaminations. RNA concentration was measured with Qubit 2.0 fluorescence meter. Moreover, RNA purity and integrity were assessed with Nanodrop spectrophotometer (Thermo Fisher Scientific, United States) and Bioanalyzer Agilent 2,100 (Agilent Technologies, United States, respectively. The 9 cDNA libraries were prepared with NEBNext®mRNA Library Prep Master Mix Set by following the manufacturer’s standard protocol. Then, the Illumina HiSeq2000 platform was used to perform *de novo* pair-end RNA sequencing by Novogene Bioinformatic Technology Co. Ltd. (Tianjin, China). After sequencing, the original sequenced data was filtered to remove low quality reads and to obtain high-quality standard reads. The *M. ternifolia* is without a reference genome, therefore high quality clean reads were assembled with Trinity software (Trinity release v2.4.0, United States) to obtain a reference sequence for succeeding analysis (Grabherr et al., 2011). The functional annotations for assembled transcripts were retrieved from publicly available protein databases including the NCBI Non-redundant nucleotide and protein database (Nr), EuKaryotic Orthologous Group (KOG), Kyoto Encyclopedia of Genes and Genomes database (KEGG), Gene Ontology (GO) Swiss-Prot, Trembl, and Pfam with Basic Local Alignment Search Tool (BLAST). The expression levels of each unigene were calculated as FPKM. The DESeq tool was used to identify DEGs among the control group and the experimental group (Anders and Huber, 2010). The identification of significant DEGs between different samples was achieved using log2 (fold change) > 1 or < − 1 and *p-value* < 0.05 as selection criteria. The FPKM of genes is first centralized and standardized and then Kmeans clustering is performed with Mfuzz software (Kumar and Futschik, 2007). The TBtools version 1.055 was utilized to make desired heatmaps (Chen et al., 2020). The pathway enrichment analysis for DEGs was retrieved as detailed previously by Kanehisa et al. (2007).

### Gene Expression Analyses With qRT-Polymerase Chain Reaction

The TransScript One-Step gDNA Removal and cDNA Synthesis SuperMix (TransGen, China) kit was utilized to synthesize the first standard cDNA for qRT-PCR. The specific primers for each gene were prepared with Oligo 7 and listed in Supplementary Table S3. For the qRT-PCR reaction mixture, QIAGEN SYBR Green PCR Kit was used with already standardized volume of each component. The *GAPDH* was used as the reference gene and the relative expression of each gene was acquired with the 2^−ΔΔCt^ method (Livak and Schmittgen, 2001). The qRT-PCR was conducted with three biological replicates and three technical replicates.

## Conclusion

In this study, 596 metabolites were identified at three developmental stages of the nutshell in *M. ternifolia*. The qualitative and quantitative metabolic data analysis revealed that phenolic acids and flavonoids were the predominant components in the nutshell. Importantly, their abundance was significantly changed during the nutshell growth periods. These results are vital for the synthesis of different by-products from the nutshell. Integration of metabolome and transcriptome analyses identified significant expression changes in phenolic acid and flavonoid pathway genes. Our results provide novel insights into the biosynthesis of polyphenols in the nutshell of *M. ternifolia*. However, functional research is essential to understand the potential genetic mechanism of phenolic acids and flavonoids metabolism in the nutshell of *M. ternifolia*.

## Data Availability

The datasets presented in this study can be found in online repositories. The names of the repository/repositories and accession number(s) can be found in the article/Supplementary Material.
