# Supplementary figures and images for: Metabolite Profiling and Transcriptome Analyses Provide Insight Into Phenolic and Flavonoid Biosynthesis in the Nutshell of Macadamia Ternifolia
Source: Front Genet. 2022 Feb 21;12:809986. doi: 10.3389/fgene.2021.809986 (PMC8899216; doi:10.3389/fgene.2021.809986)

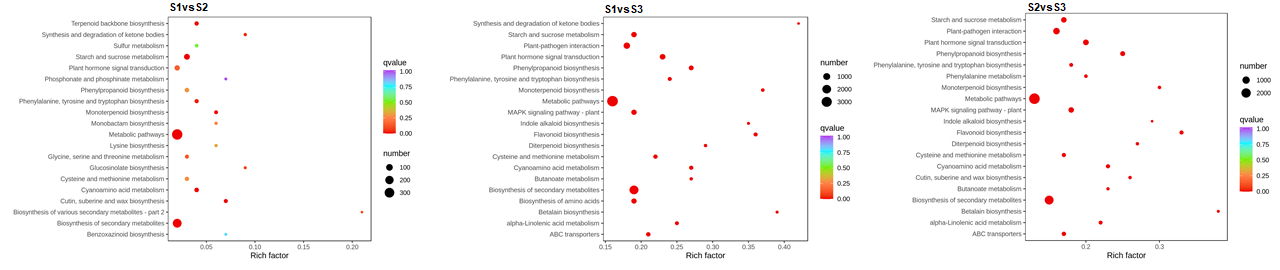

Supplement: Supplementary file 1 [file Image3.tif]

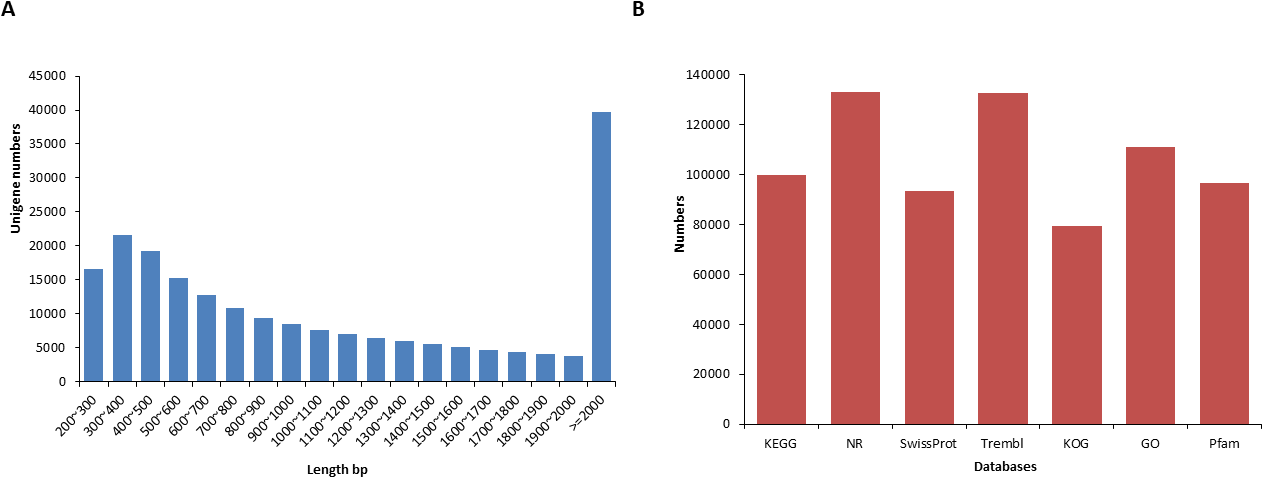

Supplement: Supplementary file 2 [file Image2.tif]

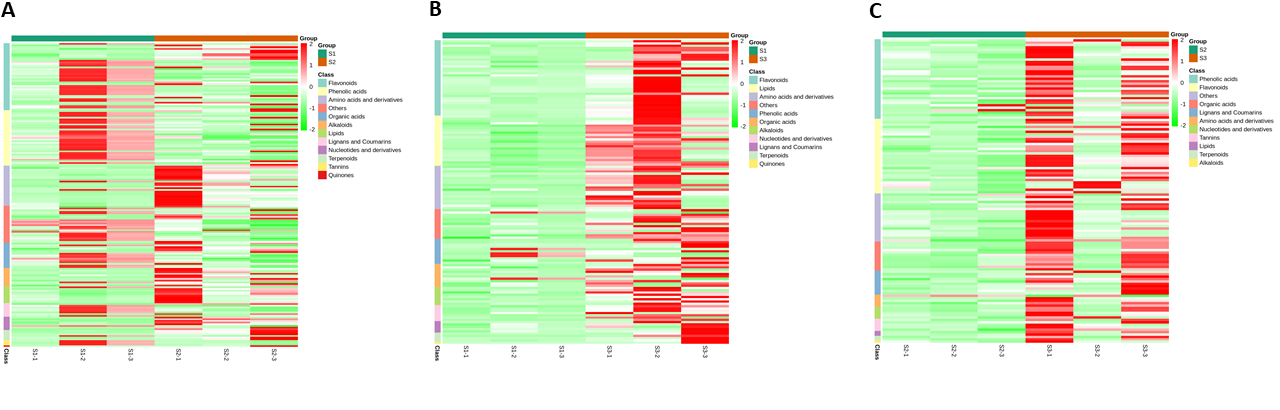

Supplement: Supplementary file 3 [file Image1.tif]
